# Structured medication review in elderly patients with chronic kidney disease to assess treatment appropriateness

**DOI:** 10.3389/fphar.2026.1915807

**Published:** 2026-09-08

**Authors:** Gloria Navarro-Aznárez, Herminia Navarro-Aznárez, María Teresa García-Ruiz, Laura Galino-Serrano, Rosalía Díaz-Royo, Alejandro Balongo-Gutiérrez, Paula Berges-Mata, Fátima Méndez-López, María Antonia Sánchez-Calavera

**Affiliations:** 1 Aragonese Healthcare Service (SALUD), Zaragoza, Spain; 2 Department of Physiatry and Nursery, Universidad de Zaragoza, Zaragoza, Spain; 3 Aragonese Primary Care Research Group, Institute for Health Research Aragón, Zaragoza, Spain; 4 Research Network on Chronicity, Primary Care and Health Promotion RD24/0005/0004, Madrid, Spain; 5 Department of Medicine, Psychiatry and Dermatology. University of Zaragoza, Zaragoza, Spain

**Keywords:** chronic kidney disease, health education, medication review, potentially inappropriate prescribing in the elderly, prevention

## Abstract

**Objective:**

to determine the proportion of patients aged 75 years or older with chronic kidney disease and an estimated glomerular filtration rate <60 mL/min/1.73 m^2^, for whom at least one medication-related treatment modification was proposed following the structured review.

**Methods:**

A single-centre, uncontrolled longitudinal study evaluating a structured medication review and the subsequent implementation of proposed treatment modifications was conducted in patients aged 75 years or older with an estimated glomerular filtration rate below 60 mL/min/1.73 m^2^. Data was obtained from laboratory systems, medical records, and electronic prescribing. Chronic medication was reviewed using a structured therapeutic appropriateness guide according to renal function. Sociodemographic variables, comorbidity, polypharmacy, need for intervention, and changes made after the treatment modifications were analysed.

**Results:**

Among the 1,937 patients aged ≥75 years registered at the primary care centre, 178 met the study eligibility criteria based on a recorded eGFR <60 mL/min/1.73 m^2^. Mean age was 86.2 years, 69.6% were women, and 88.7% were exposed to polypharmacy. At least one medication-related treatment modification was proposed for 111 patients (62.4%), with 215 individual modifications proposed overall. The therapeutic groups with the greatest need for intervention were analgesics and non-steroidal anti-inflammatory drugs, oral antidiabetic agents, and antihypertensive drugs. At follow-up, corresponding prescription changes were observed for 114 of the 215 proposed modifications (53.0%). At the patient level, at least one corresponding prescription change was observed in 64 of the 111 patients for whom one or more modifications had been proposed (57.7%).

**Conclusion:**

The structured medication review identified a substantial number of potential medication-related problems and proposed treatment modifications in older patients with reduced renal function. Approximately half of the proposed modifications were observed in the electronic prescription at follow-up. However, because the study lacked a control group, the extent to which these changes were attributable to the structured review cannot be determined.

## Background

The increase in life expectancy, especially in developed countries, has meant that in recent years older patients have become part of a population characterized by multimorbidity and the resulting polypharmacy (Otero et al., 2010), with greater exposure to adverse effects, drug interactions, prescribing cascades, *etc.* For this reason, monitoring iatrogenesis is essential.

The prevalence of chronic kidney disease (CKD) in the adult population is 9%–10%, increasing among people aged over 64 years to 21.4% and rising to 35%–40% in prevalent conditions such as hypertension (HTN) or diabetes mellitus (DM) (Bobé-Armant et al., 2019; García-Maset et al., 2022). CKD is defined as a decrease in kidney function, expressed as an estimated glomerular filtration rate (eGFR) <60 mL/min/1.73 m^2^ and/or the presence of kidney damage (albuminuria, haematuria, histological abnormalities or abnormalities on imaging tests) that persists for at least 3 months (Álvarez de Lara and García Montemayor, 2018; Bobé-Armant et al., 2019; Rovin et al., 2021; García-Maset et al., 2022). These eGFR value has different categories: G1: normal or high: ≥90 mL/min/1.73 m^2^; G2: mildly decreased: 60–89 mL/min/1.73 m^2^; G3a: mildly to moderately decreased: 45–59 mL/min/1.73 m^2^; G3b: moderately to severely decreased: 30–44 mL/min/1.73 m^2^; G4: severely decreased: 15–29 mL/min/1.73 m^2^; G5: kidney failure (Rovin et al., 2021).

The aim in managing this disease is to delay deterioration of kidney function. One of the measures to achieve this is withdrawing drugs or modifying inappropriate doses in order to avoid their associated nephrotoxicity (Álvarez de Lara and García Montemayor, 2018; García Soidán et al., 2024). Therefore, one of the roles of primary care is deprescribing in the older population, especially in frail older people (Etxeberria et al., 2018; García Soidán et al., 2024).

Systematic medication review is a structured examination of the medicines a patient takes. Its intermediate objective is to ensure and maximise their rational use, and its final objective is to ensure that the health benefits the patient obtains from pharmacological treatment are the maximum expected (Otero et al., 2010; Labrador et al., 2018).

Our working hypothesis was that structured medication review in patients aged ≥75 years with CKD stages G3a, G3b, G4 and G5 according to the KDIGO classification detects the need for treatment adjustments and that these patients would therefore benefit from such review (Rovin et al., 2021).

The primary objective was to determine the proportion of patients aged 75 years or older with chronic kidney disease and an estimated glomerular filtration rate <60 mL/min/1.73 m^2^, for whom at least one medication-related treatment modification was proposed following the structured review.

The secondary objectives were to: (1) estimate the proportion of patients aged ≥75 years who met the eGFR eligibility criterion, (2) determine the proportion of polypharmacy among the included patients, (3) identify the medications and therapeutic groups involved, and (4) assess the proportion of proposed modifications observed in the electronic prescription during follow-up.

## Methods

### Study design

This was a single-centre, uncontrolled longitudinal study evaluating a structured medication review and the subsequent implementation of proposed treatment modifications. The study population was cared for by an urban primary care team within the Aragonese Health Service. Primary care in Spain is part of the public health system, in which centres provide basic and initial care while ensuring comprehensive care and continuity throughout the patient’s life, acting as case managers, coordinators and flow regulators. It includes health promotion, health education, disease prevention, disease treatment, health maintenance and recovery, physical rehabilitation and social work (García-Serrano et al., 2019).

### Participants and eligibility criteria

Patients were eligible if they were aged ≥75 years and had an estimated glomerular filtration rate (eGFR) <60 mL/min/1.73 m^2^ in their most recent laboratory assessment available as of 31 December 2019, performed within the previous 6–12 months. When more than one eGFR measurement was available during this period, the most recent value was selected. Patients receiving renal replacement therapy, patients not covered by the public healthcare system and patients institutionalised in nursing homes within the catchment area that had their own physician were excluded. Patients were classified according to their most recent eGFR stage (Rovin et al., 2021).

### Data sources and study periods

Data were obtained from the biochemistry laboratory information system of the referral hospital, the primary care electronic health record and the electronic prescribing system. Baseline data collection and the structured medication review were conducted between 10 January and 1 March 2020. The subsequent implementation of the proposed treatment modifications was assessed between 15 November and 31 December 2021.

### Variables and outcomes

The following variables were recorded: age; sex; eGFR; eGFR category; albuminuria; number and type of comorbidities; number and type of regularly prescribed medications; presence of polypharmacy; number, type and therapeutic group of the medication-related recommendations generated; and subsequent implementation of the proposed modifications.

Polypharmacy was defined as the regular use of five or more medications (Gorostidi et al., 2018). eGFR was calculated using the Chronic Kidney Disease Epidemiology Collaboration equation. The primary outcome was the proportion of included patients for whom at least one renal-function-related medication recommendation was generated. Secondary outcomes included: (1) The proportion of patients aged ≥75 years registered at the primary care centre who met the eGFR eligibility criterion; (2) The proportion of polypharmacy among the included patients; (3) The total number and type of medication-related recommendations generated; (4) The therapeutic groups and active substances involved; (5) The proportion of recommendations subsequently implemented, assessed at both the patient and recommendation levels.

### Structured medication review

**FIGURE 1:**
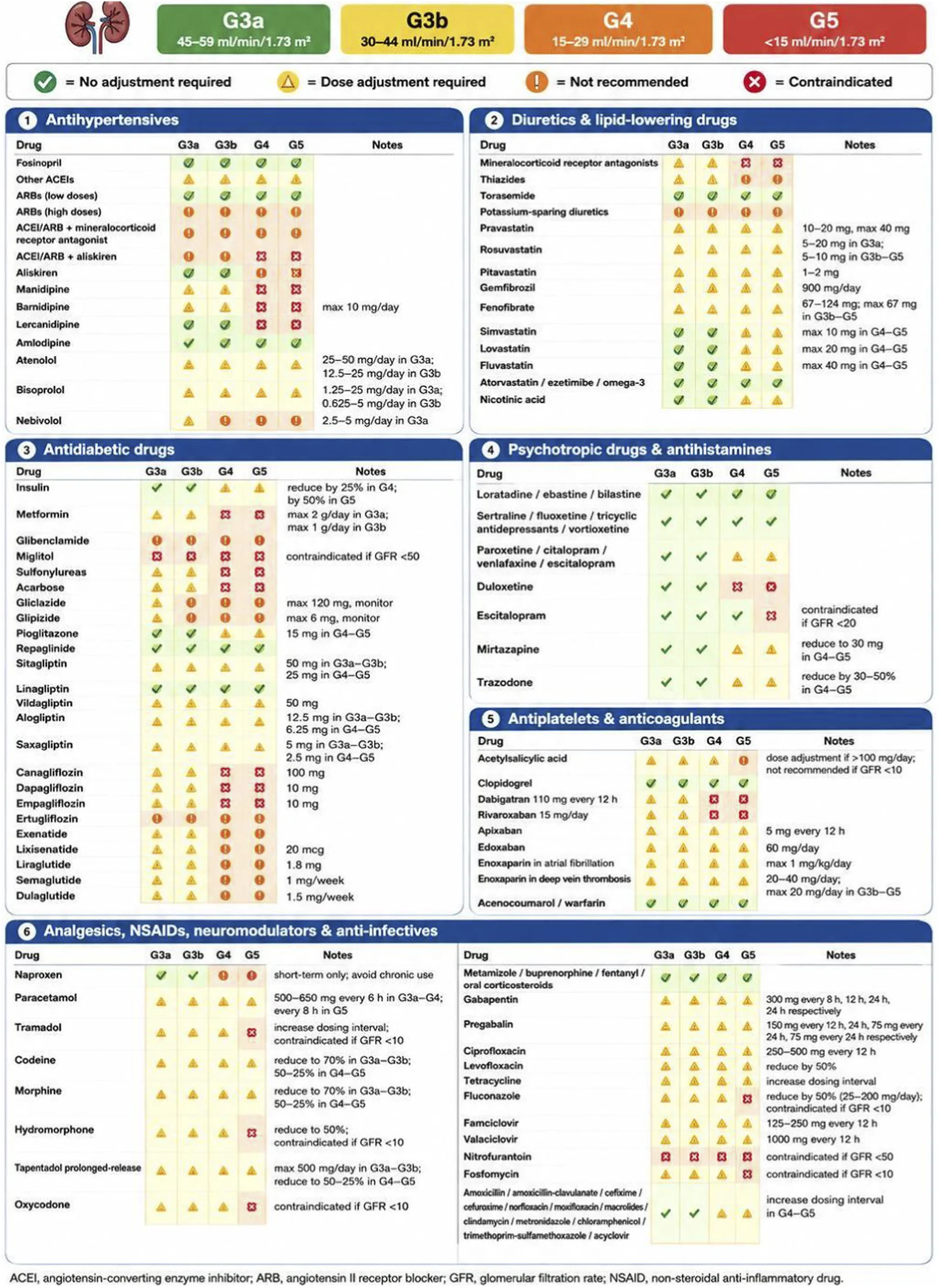
Structured medication-review guide developed by a multidisciplinary team comprising primary care physicians, nurses, and pharmacy-trained professionals, based on recommendations and clinical guidelines available as of 1 January 2020.

The structured medication-review guide presented in Figure 1 was developed by a multidisciplinary team comprising primary care physicians, nurses, and professionals with degrees in pharmacy. The guide covered commonly prescribed pharmacological groups and was based on the recommendations and clinical guidelines available as of 1 January 2020 (Álvarez de Lara and García Montemayor, 2018; Bobé-Armant et al., 2019; García Soidán et al., 2024). The full guide is provided in Supplementary Material S1. The role of the multidisciplinary team was limited to the development of the structured review tool; its members did not jointly review individual patients’ prescriptions or make patient-level prescribing decisions.

The chronic medication of each included patient was subsequently reviewed by the primary care physician responsible for their care using the electronic health record, laboratory data, and electronic prescribing system. Chronic treatment was defined as medication prescribed for more than 90 days.

The review involved two sequential stages. First, the physician applied the structured guide to identify potential discrepancies related to medication appropriateness, including treatment indication, conditions of use, and dose adjustment according to renal function. When a potential discrepancy was identified, application of the guide generated an alert and a preliminary proposed modification. For the purposes of the study, these alerts were recorded as screening findings and did not constitute final prescribing decisions or formal recommendations requiring acceptance by another professional. In a second stage, the same primary care physician reassessed each alert in the context of the patient’s complete clinical situation, including the treatment indication, comorbidities, concomitant medication, laboratory results, treatment tolerance, and, when relevant, whether the medication had been initiated or was being supervised by another specialist. Following this individual clinical assessment, the physician decided whether and when a prescription modification was appropriate.

Renal-function-related proposed modifications were classified as dose reduction, dose increase, or treatment discontinuation. Recommendations based exclusively on non-renal clinical indications, including treatment initiation, were excluded from the final renal appropriateness analysis. The final prescribing decision remained with the primary care physician responsible for the patient.

The patient-level review was based exclusively on the information available in the laboratory database, electronic health record, and electronic prescribing system. No pharmacist-led patient-level medication review, formal educational intervention, direct patient interview, or formal acceptance or rejection procedure involving a second professional was conducted as part of the study.

### Assessment of prescription changes at follow-up

Between 15 November and 31 December 2021, the electronic prescribing records were reviewed to determine whether a prescription change corresponding to each preliminary proposed modification was evident at follow-up. At the patient level, a corresponding change was considered observed when at least one of the proposed modifications was reflected in the electronic prescription. At the medication level, the proportion was calculated by dividing the number of proposed modifications with a corresponding prescription change observed at follow-up by the total number of proposed modifications. No formal acceptance or rejection decision was recorded, and the reasons why individual changes were not observed were not systematically documented.

### Statistical analysis

A descriptive and bivariate analysis were performed. Continuous variables were assessed for normality through visual inspection of histograms and Q–Q plots and using the Shapiro–Wilk test. Normally distributed variables are presented as mean ± standard deviation and were compared between independent groups using Student’s t-test. Non-normally distributed variables are presented as median and interquartile range and were compared using the Mann–Whitney U test. Categorical variables are reported as frequencies and percentages and were compared using the chi-square test or Fisher’s exact test, as appropriate. All statistical analyses were performed using IBM SPSS Statistics, version 21.0, and statistical significance was set at p < 0.05.

### Ethical considerations

The study was approved by the Research Ethics Committee of Aragon (CEICA, PI20/027). Data confidentiality: data were dissociated and used only for the purposes of the study. Patient confidentiality was guaranteed in accordance with Organic Law 3/2018 of 5 December on Personal Data Protection and Guarantee of Digital Rights. The study was conducted in accordance with the Declaration of Helsinki.

## Results

The analytical dataset included 178 patients. Mean age was 86.2 ± 5.2 years, and 124 patients (69.7%) were women. The median eGFR was 38.2 mL/min/1.73 m^2^ (IQR 33.3–42.1), with values ranging from 15.7 to 45.9 mL/min/1.73 m^2^. Five patients (2.8%) were classified as G3a, 143 (80.3%) as G3b and 30 (16.9%) as G4. Polypharmacy was present in 158 patients (88.8%).

Microalbuminuria had been requested in only 23% of patients; among these, the most frequent category was A1 (<30 mg/g), at 53.7%, while 14.6% of patients were in category A3 (>299 mg/g).

At least one medication-related treatment modification was proposed for 111 patients (62.3%). Compared with patients for whom no modification was proposed, these patients had a lower median eGFR, a greater number of comorbidities and medications, and a higher prevalence of hypertension, diabetes, hyperlipidaemia, musculoskeletal disease, psychiatric disorders and polypharmacy. Detailed results are shown in Table 1.

**TABLE 1:** Baseline characteristics of study patients.

Variable	Total (n = 178)	Need for intervention n = 111 (62.3%)	No need for intervention n = 67 (37.7%)	p
Sex, n (%)				
Men	54 (30.3)	33 (29.7)	21 (31.3)	0.901
Women	124 (69.6)	78 (70.2)	46 (68.6)	
Age, years, mean ± SD	86.2 ± 5.2	86.3 ± 5.2	86.1 ± 5.1	0.736
Open CKD episode, n (%)	143 (80.3)	87 (78.3)	56 (83.5)	0.079
eGFR, ml/min/1.73 m^2^, (median, IQR)	(38.2, 8.8)	(37, 9.18)	(39.9, 7.9)	0.008
CKD stages, n (%)				
G3A	5 (2.8)	3 (2.7)	2 (2.9)	0.136
G3B	143 (80.3)	84 (75.6)	59 (88)	
G4	30 (16.9)	24 (21.6)	6 (8.9)	
Comorbidities				
Number of comorbidities, (median, IQR)	(6.0, 3.0)	(7.0, 4.0)	(5.0, 3.0)	<0.001
Hypertension, n (%)	140 (78.6)	97 (87.3)	43 (64.1)	0.001
Diabetes mellitus, n (%)	68 (38.2)	51 (45.9)	17 (25.3)	0.004
Hyperlipidaemia, n (%)	89 (50.0)	64 (57.6)	25 (37.3)	0.031
COPD, n (%)	27 (15.1)	16 (14.4)	11 (16.4)	0.768
Musculoskeletal diseases, n (%)	113 (63.4)	82 (73.8)	31 (46.2)	0.001
Neurological diseases, n (%)	43 (24.1)	27 (24.3)	16 (23.8)	0.878
Digestive diseases, n (%)	33 (18.5)	23 (20.7)	10 (14.9)	0.301
Endocrine diseases, n (%)	43 (24.1)	25 (22.5)	18 (26.8)	0.836
Cardiovascular diseases, n (%)	101 (57.0)	67 (60.3)	34 (50.7)	0.312
Psychiatric disorders, n (%)	68 (38.2)	50 (45)	18 (26.8)	0.027
Treatment				
Polypharmacy (≥5 drugs),n (%)	158 (88.7)	105 (94.5)	53 (79.1)	0.009
Number of drugs, (median, IQR)	(10.0, 6.7)	(11.6, 5.0)	(7.5, 5.0)	<0.001

Data are presented as n (%), mean ± SD, or (median, IQR), as indicated.

CKD, chronic kidney disease; COPD, chronic obstructive pulmonary disease; eGFR, estimated glomerular filtration rate; IQR, interquartile range; SD, standard deviation; CKD stages, G3a, eGFR 45–59 mL/min/1.73 m^2^; G3b, 30–44 mL/min/1.73 m^2^; and G4, 15–29 mL/min/1.73 m^2^.

### Characteristics of proposed and implemented treatment modifications

Of the 178 patients, 111 (%) required treatment modifications. A total of 215 interventions were needed, with a mean of 01.9 proposed modifications per patient with at least one identified modification.

The types of proposed treatment modifications were divided into four categories: dose reduction, dose increase, drug withdrawal and introduction of a non-prescribed drug. Dose reduction was required in % of interventions (195/215), dose increase in % (1/215), drug withdrawal in % (35/215). No treatment-initiation recommendations were included in the final renal-function-related analysis. At the patient level, implementation indicates that at least one proposed modification was observed in the electronic prescription during follow-up.

A total of 215 individual medication-related modifications were proposed across the 111 patients identified by the structured review. The largest numbers of proposed modifications concerned paracetamol (n = 36), metformin (n = 14), olmesartan and mirtazapine (n = 13 each), acetylsalicylic acid (n = 12), NSAIDs (n = 10), and fibrates (n = 10). Detailed results by therapeutic group and active substance are presented in Table 2.

**TABLE 2:** Proposed medication-related modifications and corresponding prescription changes observed at follow-up, by medication.

Prescribed drugs	Total of prescription	Proposed modifications n (% of prescriptions)	Corresponding prescription changes observed at follow-up n (% of proposed modifications)
Analgesics			
Paracetamol	122	36 (29.5%)	28 (77.7%)
Tramadol	39	7 (17.9%)	5 (71.4%)
Tapentadol	11	3 (27.2%)	3 (100%)
NSAIDs	10	10 (100%)	10 (100%)
Oral antidiabetic drugs			
Metformin	31	14 (45.1%)	11 (78.5%)
Sulfonylureas	6	1 (16.6%)	0 (0%)
Linagliptin	24	0 (0%)	0 (0%)
Sitagliptin	6	5 (83.3%)	2 (40%)
Vildagliptin	6	4 (66.6%)	2 (50%)
Saxagliptin	1	1 (100%)	0 (0%)
SGLT2 inhibitors	3	2 (66.6%)	0 (0%)
Pioglitazone	3	1 (33.3%)	0 (0%)
Antihypertensives			
Olmesartan	27	13 (48.1%)	5 (38.4%)
Other ARBs	60	14 (23.3%)	6 (42.8%)
ACE inhibitors	41	8 (19.5%)	2 (25%)
CCBs	28	1 (3.5%)	1 (100%)
Bisoprolol	5	2 (40%)	0 (0%)
Diuretics			
Hydrochlorothiazide	73	6 (8.2%)	2 (33.3%)
Spironolactone	21	6 (28.5%)	3 (50%)
Chlorthalidone	1	1 (100%)	0 (0%)
Indapamida	6	1 (16.6%)	0 (0%)
Antiplatelet drugs			
ASA	25	12 (48%)	5 (41.6%)
Pentoxifylline	2	1 (50%)	0 (0%)
Anticoagulants			
Apixaban	14	5 (35.7%)	0 (0%)
Rivaroxaban	6	3 (50%)	0 (0%)
Edoxaban	4	2 (50%)	0 (0%)
Dabigatran	2	1 (50%)	0 (0%)
Acenocoumarol	19	0 (0%)	0 (0%)
Lipid-lowering drugs			
Statins (atorvastatine excluded)	47	5 (10.6%)	5 (100%)
Fibrates	10	10 (100%)	10 (100%)
Antihistamines	22	4 (18.1%)	3 (75%)
Antidepressants			
Mirtazapine	13	13 (100%)	0 (0%)
Trazodone	9	1 (11.1%)	1 (100%)
Duloxetine	3	1 (33.3%)	1 (100%)
Other			
Allopurinol	9	8 (88.8%)	4 (50%)
Gabapentin	5	3 (60%)	1 (33.3%)
Cholecalciferol	10	7 (70%)	4 (57.1%)
Digoxin	1	1 (100%)	0 (0%)
Colchicine	1	1 (100%)	0 (0%)
Levetiracetam	1	1 (100%)	0 (0%)

ACE, angiotensin-converting enzyme; ARB, angiotensin II, receptor blocker; ASA, acetylsalicylic acid; CCB, calcium-channel blocker; DOAC, direct oral anticoagulant; DPP-4, dipeptidyl peptidase-4; NSAID, non-steroidal anti-inflammatory drug; SGLT2, sodium-glucose cotransporter 2.

### Changes observed at follow-up

At follow-up, at least one corresponding prescription change was observed in 64 of the 111 patients for whom one or more modifications had been proposed (57.7%). At the medication level, 114 of the 215 proposed modifications were evident in the electronic prescribing records at follow-up (53.0%) (Table 3).

**TABLE 3:** Summary of proposed and implemented treatment modifications.

Level of analysis	Proposed	Implemented at follow-up	Proportion with a corresponding change observed at follow-up
Patients with ≥1 proposed modification	111	64	57.6%
Individual medication-related modifications	215	114	53%

At the patient level, a corresponding change was considered observed when at least one proposed medication modification was evident in the electronic prescription at follow-up. At the medication level, the proportion was calculated by dividing the number of proposed modifications evident at follow-up by the total number of individual modifications proposed.

Among medications with at least 10 proposed modifications, corresponding changes were observed for all NSAID- and fibrate-related proposals, for 11 of 14 metformin-related proposals (78.6%), and for 28 of 36 paracetamol-related proposals (77.8%). No corresponding prescription change was observed at follow-up for the mirtazapine-related or DOAC-related proposals. Eleven DOAC prescriptions were flagged for review because the prescribed dose was not concordant with the renal-function-related dosing criteria applicable to the corresponding active substance. Full drug-specific results are provided in Table 3. In addition, eight patients (4.5%) had concomitant prescriptions for an NSAID, a renin–angiotensin system inhibitor, and a diuretic, representing potential exposure to the triple-whammy combination.

## Discussion

The proportion of CKD in our study was very low, and although some Spanish studies also reported low prevalence (Frutos Bernal et al., 2011; Llisterri et al., 2021), in Spain the prevalence of this disease (at any stage) in the general population is 15.1% according to the results of the Study on Nutrition and Cardiovascular Risk in Spain (ENRICA), similar to the 14.4% of the population cared for in primary care in the IBERICAN study (Identification of the Spanish Population at Cardiovascular and Renal Risk). Both studies show increases in prevalence with age and with cardiovascular disease (Peña et al., 2003; Martínez-Arnau et al., 2016). However, it should be borne in mind that we studied only older people aged 75 years or older from a single health centre, unlike the aforementioned studies (ENRICA or IBERICAN). Incomplete ascertainment of renal function, under-recording and selection bias may therefore have contributed to the lower proportion observed. Moreover, the small number of patients classified as G3a suggests that the study cohort may not reflect the expected distribution of CKD stages in the source population.

For the diagnosis and screening of CKD, eGFR and the albumin/creatinine ratio (ACR) must be determined in a urine sample. However, our study found that this determination had been requested in only 23.0% of patients, a finding also reported in the literature, which describes that many patients at risk of CKD (hypertension, type 2 DM, among others) do not have periodic ACR monitoring (García-Maset et al., 2022). It is therefore very important to study and identify the possible reasons for our low results in order to correct them, as this is a particularly frail population that must be properly diagnosed and cared for.

In agreement with the literature consulted (García Soidán, 2018; Carbayo García et al., 2014; Franch-Nadal et al., 2022), the most frequent health problems in patients exposed to polypharmacy are hypertension, musculoskeletal diseases, cardiovascular diseases, dyslipidaemia and diabetes.

When analysing the results by therapeutic group, overall, 62.4% of patients were using medicines at doses inappropriate for their kidney disease stage, a figure similar to that in the literature consulted. However, those references study specific diseases and single therapeutic groups, without studying several groups as in our case (Bannwarth and Péhourcq, 2003). A recent multicentre study conducted in Spanish community pharmacies identified at least one potentially inappropriate prescription in 90 of 215 patients with an eGFR <60 mL/min/1.73 m^2^. Although its setting, selection criteria and review procedure differed from those of our study, it also showed that only a limited proportion of the proposed medication changes was subsequently implemented. These findings reinforce the potential value of structured medication review while highlighting the importance of effective communication and follow-up procedures (Escribá-Martí et al., 2025).

Analgesics, oral antidiabetic agents, and antihypertensive drugs were among the therapeutic groups most frequently involved in the proposed treatment modifications. Analgesics and NSAIDs were analysed separately because of their different pharmacological profiles and renal safety considerations. Paracetamol was the most frequently prescribed analgesic. This finding is clinically relevant because NSAID use has been associated with renal impairment in older adults, making non-NSAID analgesic strategies particularly important in patients with CKD (Henry et al., 1997).

Metamizole was prescribed in 12.0% of patients. Although no renal dose adjustment was indicated according to the guide used in this study, its long-term use may require clinical monitoring because of potential adverse effects. NSAIDs and cyclooxygenase-2 inhibitors were prescribed in a relatively small proportion of patients; nevertheless, their use is clinically relevant in CKD because they may reduce renal perfusion and increase the risk of acute kidney injury and hyperkalaemia, particularly when combined with a renin–angiotensin system inhibitor and a diuretic (Camin et al., 2015). At follow-up, the NSAID was no longer present in the electronic prescription in all 10 cases for which discontinuation had been proposed, thereby removing the NSAID component of the potential triple-whammy combination (Ferrat et al., 2021).

Oral antidiabetic agents were among the therapeutic groups most frequently involved in the proposed medication modifications. Among patients receiving metformin, 14 of 31 prescriptions (45.2%) were flagged for modification according to renal function, consistent with the importance of adjusting metformin treatment in patients with reduced eGFR. Dipeptidyl peptidase-4 inhibitors were analysed by active substance because their renal dose-adjustment requirements differ. In the present study, proposed modifications involved sitagliptin, vildagliptin, and saxagliptin, whereas no renal dose adjustment was proposed for linagliptin (Bannwarth and Péhourcq, 2003; Franch-Nadal et al., 2022).

Sulfonylureas were infrequently prescribed in this cohort. Nevertheless, their use warrants particular caution in frail older adults because of the risk of hypoglycaemia (García Soidán, 2018). Only three patients were receiving a sodium–glucose cotransporter-2 inhibitor, and treatment discontinuation was proposed in two cases according to the renal-function recommendations applicable when the structured guide was developed in January 2020. Since recommendations for SGLT2 inhibitors have subsequently evolved, these findings should be interpreted in their historical context rather than applied to current prescribing practice (Nuffield Department of Population Health Renal Studies Group and SGLT2 inhibitor Meta-Analysis Cardio-Renal Trialists’ Consortium, 2022).

Among antihypertensive agents, angiotensin II receptor blockers were prescribed more frequently than angiotensin-converting enzyme inhibitors, and olmesartan accounted for most of the proposed modifications within this therapeutic group (Montemayor et al., 2024; Agencia Española de Medicamentos y Productos Sanitarios, 2025). However, the appropriateness of renin–angiotensin system inhibitor treatment should not be assessed solely on the basis of eGFR. Clinical indication, albuminuria, serum potassium, blood pressure, treatment tolerance, and the patient’s overall clinical condition should also be considered. The KDIGO 2024 guideline supports the continued use of angiotensin-converting enzyme inhibitors or angiotensin II receptor blockers in many patients with CKD when clinically indicated, with appropriate monitoring for adverse effects (Stevens et al., 2024).

Direct oral anticoagulants were prescribed in 26 patients (14.6%). In the revised analysis, only proposed dose modifications for which renal function formed part of the applicable dosing criterion were retained. Recommendations based exclusively on age, body weight, clinical indication, bleeding risk, concomitant treatment or other non-renal considerations were excluded, as was the recommendation to initiate anticoagulation for an untreated clinical indication. DOAC dosing remains complex because the applicable criteria differ according to the specific active substance and may incorporate renal function together with other patient characteristics. Previous studies have reported potentially inappropriate DOAC dosing in older patients, which may be associated with thromboembolic (Beyer-Westendorf et al., 2021) or bleeding complications (Dillinger et al., 2018; Cavillon Decaestecker et al., 2021). No corresponding prescription change was observed at follow-up for the renal-function-related DOAC proposals. However, this finding should not be interpreted as evidence that the proposals were explicitly rejected by the prescribers. The reasons why changes were not observed were not systematically recorded, and the delayed follow-up assessment may also have failed to detect modifications that had been implemented and subsequently reversed before the electronic prescribing records were reviewed.

### Limitations

The principal limitation of this study is the absence of a concurrent control group. Consequently, the prescription changes observed during follow-up cannot be attributed exclusively to the structured medication review, as some modifications may have occurred as part of routine clinical care or in response to changes in the patients’ clinical status. Conversely, the absence of an observed prescription change should not be interpreted as evidence that a proposed modification was explicitly rejected by the prescriber. The reasons why individual modifications were not observed at follow-up were not systematically recorded and therefore cannot be determined from the available data.

The long interval between the initial medication review and the follow-up assessment further limited the interpretation of the findings. Because electronic prescribing records were reviewed only between 15 November and 31 December 2021, the study could not determine when each prescription modification occurred. A proposed change may have been implemented earlier and subsequently reversed before the follow-up assessment, in which case it would not have been detected. Similarly, renal function, clinical status, treatment indication or therapeutic needs may have changed during the interval, potentially altering the appropriateness of the original proposal.

The study was conducted in a single primary care centre and relied on routinely collected electronic clinical, laboratory and prescribing data, which limits its external validity and may have introduced incomplete recording or misclassification. The proportion of registered patients who met the eligibility criteria was lower than the prevalence of CKD reported in older Spanish populations. However, this figure should not be interpreted as a population prevalence estimate because the denominator included all registered patients aged ≥75 years, whereas eligibility depended on the availability of a recent eGFR measurement and the application of the study exclusion criteria. Incomplete ascertainment of renal function, under-recording and selection bias may therefore have contributed to the lower proportion observed. In addition, the unusual distribution of eGFR categories, with very few patients classified as G3a, suggests that the study sample may not reflect the expected distribution of CKD stages in the source population.

Albuminuria data were incomplete, limiting the characterisation of CKD severity and risk. The study also did not assess clinical outcomes such as adverse drug events, hospital admissions, changes in renal function, bleeding, thromboembolic events or mortality. Therefore, the clinical consequences of the proposed and subsequently observed prescription modifications remain unknown.

Finally, follow-up occurred in the context of the COVID-19 pandemic, during which healthcare services underwent substantial reorganisation and routine management of chronic conditions may have been disrupted. Although this context may have influenced follow-up and prescription management, its specific effect cannot be quantified in the absence of a comparison group. Overall, the findings should be interpreted as descriptive and hypothesis-generating rather than as evidence of a causal effect of the structured medication review.

### Implications for practice and future research

Despite these limitations, the study identified a substantial number of potential medication-related problems in older patients with reduced renal function. Structured medication review requires additional professional time and access to updated clinical and laboratory information. Its integration into routine practice could be supported by training strategies and electronic prescribing-assistance systems linked to the patient’s medical record, including alerts for potentially inappropriate prescriptions and renal dose-adjustment requirements (Diz-Lois Martínez et al., 2012; Salech et al., 2016; Stevens et al., 2024).

## Conclusion

The proportion of patients aged >75 years with GFR <60 mL/min/1.73 m^2^ was very low compared with other studies. The percentage of patients for whom at least one modification was proposed was high, close to two-thirds of the study population.

The profile of patients for whom at least one modification was proposed was that of patients with greater comorbidity and more drugs in their treatment regimen, especially for hypertension, DM, dyslipidaemia, musculoskeletal disorders, cardiovascular diseases and psychiatric disorders.

Only half of the proposed medication modifications were carried out after the treatment modifications, the most frequent involving paracetamol, NSAIDs, fibrates and metformin. However, it is notable that no intervention was carried out for DOACs. It is necessary to continue promoting and training professionals so that medication review in patients exposed to polypharmacy is integrated into patient care, becomes part of routine practice and can be carried out in a structured way, especially in patients with CKD.

## Data Availability

The raw data supporting the conclusions of this article will be made available by the authors, without undue reservation.

## Glossary

A1: normal to mildly increased albuminuria category
A3: severely increased albuminuria category
ACE: angiotensin-converting enzyme
ACEI: angiotensin-converting enzyme inhibitor
ACR: albumin-to-creatinine ratio
ARB: angiotensin II receptor blocker
ASA: acetylsalicylic acid
BZD: benzodiazepine
CCB: calcium-channel blocker
CEICA: Research Ethics Committee of Aragón
CI: contraindicated
CKD: chronic kidney disease
CKD-EPI: Chronic Kidney Disease Epidemiology Collaboration equation
COPD: chronic obstructive pulmonary disease
COX-2: cyclooxygenase-2
DA: dose adjustment
DM: diabetes mellitus
DOAC: direct oral anticoagulant
DPP-4: dipeptidyl peptidase-4
eGFR: estimated glomerular filtration rate
ENRICA: Study on Nutrition and Cardiovascular Risk in Spain
GAIAP: Aragonese Primary Care Research Group
GFR: glomerular filtration rate
HLP: hyperlipidaemia
HTN: hypertension
IBERICAN: Identification of the Spanish Population at Cardiovascular and Renal Risk
IIS Aragón: Aragón Health Research Institute
KDIGO: Kidney Disease Improving Global Outcomes
NA: no adjustment
NR: not recommended
NSAID: non-steroidal anti-inflammatory drug
OMI-AP: primary care electronic medical record system
SARS-CoV-2: severe acute respiratory syndrome coronavirus 2
SD: standard deviation
SGLT2: sodium–glucose cotransporter 2
SPSS: Statistical Package for the Social Sciences
