# Supplementary material for: Structured medication review in elderly patients with chronic kidney disease to assess treatment appropriateness
Source: Front Pharmacol. 2026 Sep 8;17:1915807. doi: 10.3389/fphar.2026.1915807 (PMC13593841; doi:10.3389/fphar.2026.1915807)
Supplement: Supplementary file 1 [file Supplementaryfile1.docx]

Suplementary 1. Guide of recommendations on commonly used pharmacological groups, adapted from various sources as of 1 January 2020.

| **Chronic kidney disease stage** | | **G3a** | **G3b** | **G4** | **G5** |
| --- | --- | --- | --- | --- | --- |
| **Glomerular filtration rate (ml/min/1.73 m²)** | | **45–59** | **30–44** | **15–29** | **<15** |
| **Antihypertensives** | | | | | |
| ACE inhibitors | Fosinopril | NA | | | |
|  | Other | DA | | | |
| Calcium-channel blockers | Manidipine | DA | | CI | |
|  | Barnidipine | DA 10 mg/day | | CI | |
|  | Lercanidipine | NA | | CI | |
|  | Amlodipine | NA | | | |
| ARBs | Low doses | NA | | | |
|  | High doses | NR | | | |
|  | Losartan, valsartan, irbesartan | NA | | | |
| ACEI or ARB + mineralocorticoid receptor antagonist | | NR | | | |
| ACEI or ARB + aliskiren | | NR | | CI | |
| Aliskiren | | NA | | NR | |
| Beta-blockers | Carvedilol, labetalol, metoprolol, propranolol | NA | | | |
|  | Atenolol | DA 25–50 mg/day | | DA 12.5–25 mg/day | |
|  | Bisoprolol | DA 1.25–25 mg/day | | DA 0.625–5 mg/day | |
|  | Nebivolol | DA 2.5–5 mg/day | | NR | |
| **Diuretics** | | | | | |
| Mineralocorticoid receptor antagonists | | DA | | CI | |
| Thiazides | | DA | | NR | |
| Torasemide | | NA | | | |
| Potassium-sparing diuretics | | NR | | | |
| **Lipid-lowering drugs** | | | | | |
| Pravastatin | | DA 10–20 mg; Max 40 mg | | | |
| Rosuvastatin | | DA 5–20 mg | | | DA 5–10 mg |
| Pitavastatin | | DA 1–2 mg | | | |
| Gemfibrozil | | DA 900 mg | | | |
| Fenofibrate | | DA 67–124 mg | | DA Max 67 mg | |
| Simvastatin | | NA | | DA Max 10 mg | |
| Lovastatin | | NA | | DA Max 20 mg | |
| Fluvastatin | | NA | | | DA Max 40 mg |
| Atorvastatin, ezetimibe, omega 3 | | NA | | | |
| Nicotinic acid | | NA | | | DA |
| **Antidiabetic drugs** | | | | | |
| Insulin | | NA | | DA ↓ by 25% | DA ↓ by 50% |
| Metformin | | DA 2 g | DA 1 g | CI | |
| Glibenclamide | | NR | | | |
| Miglitol | | CI if GFR is <50 | | | |
| Sulfonylureas | | DA | | CI | |
| Acarbose | | DA | | CI | |
| Gliclazide | | DA 120 mg, monitoring | | NR | |
| Glipizide | | DA 6 mg, monitoring | | NR | |
| Pioglitazone | | NA | | | DA 15 mg |
| Repaglinide | | NA | | | |
| Sitagliptin | | DA 50 mg | | DA 25 mg | |
| Linagliptin | | NA | | | |
| Vildagliptin | | DA 50 mg | | | |
| Alogliptin | | DA 12.5 mg | | DA 6.25 mg | |
| Saxagliptin | | DA 5 mg | DA 2.5 mg | | |
| Canagliflozin | | DA 100 mg | | CI | |
| Dapagliflozin | | DA 10 mg | CI | | |
| Empagliflozin | | DA 10 mg | CI | | |
| Ertugliflozin | | NR | | | |
| Exenatide | | DA 10 | | NR | |
| Lixisenatide | | DA 20 mcg | | NR | |
| Liraglutide | | DA 1.8 mg | | NR | |
| Semaglutide | | DA 1 mg/week | | NR | |
| Dulaglutide | | DA 1.5 mg/week | | NR | |
| **Antihistamines** | | | | | |
| Loratadine, ebastine, bilastine | | NA | | | |
| **Antidepressants** | | | | | |
| Sertraline, fluoxetine, tricyclic antidepressants, vortioxetine | | NA | | | |
| Paroxetine, citalopram, venlafaxine, escitalopram | | NA | | DA | |
| Duloxetine | | NA | | CI | |
| Escitalopram | | NA | | CI if GFR is <20 | |
| Mirtazapine | | NA | DA, ↓ to 30 mg | | |
| Trazodone | | NA | DA, ↓ by 30–50% | | |
| **Antiplatelet drugs** | | | | | |
| Acetylsalicylic acid | | DA if >100 mg/day | | | NR if GFR is <10 |
| Clopidogrel | | NA | | | |
| **Anticoagulants** | | | | | |
| Dabigatran 110 mg/12 h | | DA 110 mg/12 h | | CI | |
| Rivaroxaban 15 mg/24 h | | DA 15 mg/24 h | | CI | |
| Apixaban | | DA 5 mg/12 h | | | |
| Edoxaban | | DA 60 mg/24 h | | | |
| Enoxaparin | in atrial fibrillation | DA Max 1 mg/kg/24 h | | | |
|  | in deep vein thrombosis | DA 20–40 mg/24 h | | DA Max 20 mg/day | |
| Acenocoumarol, warfarin | | NA | | | |
| **Non-steroidal anti-inflammatory drugs** | | | | | |
| Naproxen | | NA if used for more than 7 days, monitor kidney function | | | |
| **Analgesics** | | | | | |
| Paracetamol | | DA 650–500 mg/6 h | | | DA 650–500 mg/8 h |
| Tramadol | | DA, increase dosing intervals | | | CI if GFR is <10 |
| Codeine | | DA ↓ to 70% | | DA ↓ to 50–25% | |
| Morphine | | DA ↓ to 70% | | DA ↓ to 50–25% | |
| Hydromorphone | | DA to 50% | | | CI if GFR is <10 |
| Prolonged-release tapentadol | | DA, Max 500 mg | | DA ↓ to 50–25% | |
| Oxycodone | | DA | | | CI if GFR is <10 |
| Metamizole, buprenorphine, fentanyl, oral corticosteroids | | NA | | | |
| **Neuromodulators** | | | | | |
| Gabapentin | | DA 300 mg/8 h | | DA 300 mg/12 h | DA 300 mg/24 h |
| Pregabalin | | DA 150 mg/12 h | | DA 150 mg/24 h | DA 75 mg/24 h |
| **Anti-infectives** | | | | | |
| Ciprofloxacin | | DA 250–500 mg/12 h | | | |
| Levofloxacin | | DA ↓ by 50% | | | |
| Tetracycline | | DA, increase dosing intervals | | | |
| Fluconazole | | DA ↓ by 50%, 25–200 mg/day | | | CI if GFR is <10 |
| Famciclovir | | DA 125–250 mg/12 h | | | |
| Valaciclovir | | DA 1000 mg/12 h | | | |
| Nitrofurantoin | | CI if GFR is <50 | | | |
| Fosfomycin | | DA | | | CI if GFR is <10 |
| Amoxicillin, amoxicillin-clavulanic acid, cefixime, cefuroxime, norfloxacin, moxifloxacin, macrolides, clindamycin, metronidazole, chloramphenicol, trimethoprim-sulfamethoxazole, aciclovir | | NA | | DA, increase dosing intervals | |

NA = no adjustment; DA = dose adjustment; NR = not recommended; CI = contraindicated; ACEI = angiotensin-converting enzyme inhibitors; ARB = angiotensin II receptor blockers; ↓ = decrease; ↑ = increase; Max = maximum
